# Synchronous Primary Endometrial and Ovarian Cancers: Trends and Outcomes of the Rare Disease at a South Asian Tertiary Care Cancer Center

**DOI:** 10.7759/cureus.9163

**Published:** 2020-07-13

**Authors:** Nazish Khalid, Faizan Ullah, Hania Zafar, Abdul Wahid Anwer, Taskheer Abbas, Osama Shakeel, Muhammad Faisal, Tabinda Sadaf, Aamir Ali Syed

**Affiliations:** 1 Surgical Oncology, Shaukat Khanum Memorial Cancer Hospital and Research Centre, Lahore, PAK; 2 Obstetrics and Gynecology, The Indus Hospital, Lahore, PAK; 3 Obstetrics and Gynecology, Nishtar Medical College and Hospital, Multan, PAK; 4 Radiation Oncology, Shaukat Khanum Memorial Cancer Hospital and Research Centre, Lahore, PAK; 5 Head and Neck Diseases, Evangelisches Krankenhaus, Vienna, AUT; 6 Oncology, Shaukat Khanum Memorial Cancer Hospital and Research Centre, Lahore, PAK

**Keywords:** synchronous primary cancers, endometrial cancers, ovarian cancer

## Abstract

Background and objective

The incidence of synchronous primary endometrial and ovarian cancer is uncommon and poses a diagnostic challenge to the treating physician about their origin as either primary or metastasis. The purpose of this study was to evaluate the clinicopathological behavior, treatment modality-related outcomes, and prognosis related to primary endometrial and ovarian cancers at a tertiary care referral center in South Asia.

Methods

We retrospectively analyzed 30 patients with synchronous ovarian and endometrial cancers treated at Shaukat Khanum Memorial Cancer Hospital and Research Centre in Lahore, Pakistan from January 2005 to August 2017.

Results

The median age of the patients at the time of diagnosis was 51 years (range: 25-72 years). The common presenting symptoms were irregular uterine bleeding (30%), post-menopausal bleeding (26.7%), abdominal mass (16.7%), and abdominal pain (26.7%). Endometrial adenocarcinoma type was the most common histological variant found among the participants: 90% (n=27) of uterine and 56.7% (n=17) of ovarian cancers. All patients underwent surgical intervention. Among them, 25 patients received platinum-based adjuvant chemotherapy, four received neoadjuvant chemotherapy, and 18 received adjuvant radiotherapy. The early-stage group [International Federation of Gynecology and Obstetrics (FIGO) stage I and II] had a more favorable prognosis than the advanced stage group (FIGO stages III and IV).

Conclusion

Based on our findings, patients with synchronous primary endometrial and ovarian cancers have better overall survival rates than patients with single primary ovarian or endometrial cancers. Also, synchronous primary endometrial and ovarian cancer endometroid types have better overall survival than patients with non-endometrioid or mixed histologic types.

## Introduction

Synchronous malignancies in the female genital tract are very rare entities (0.5-1.7%). Among them, synchronous endometrial and ovarian tumors are the most common types of malignancy, with a frequency of 5% among endometrial and 10% among ovarian primary tumors [1,2]. Clinical presentation of these women is indistinct, and extensive pathological evaluation has to be performed to distinguish synchronous endometrial and ovarian malignancies from the metastatic disease. Several studies have been published about the diagnostic criteria for multiple primary tumors with certain limitations [3,4]. However, Scully et al. have described illustrative clinicopathological details and classified them into three groups: (a) primary endometrial tumors with ovarian metastasis, (b) primary ovarian tumors with endometrial dissemination, and (c) synchronous endometrial and ovarian malignancies [5]. Published literature has reported better outcomes in the synchronous disease category as compared to the groups with a single primary having metastatic disease [6-8]. Zaino et al. have reported five- and 10-year survival rates of 86% and 80% for synchronous and single primary patients, respectively [7]. Young age, obesity, pre-menopausal status, and nulliparity were the distinct features among this rare entity. Similarly, patients with a synchronous endometroid variant of ovarian or endometrial tumors have demonstrated favorable outcomes compared to other patient groups (119 vs. 48 months) [9]. The objective of this study was to evaluate the clinicopathological features, treatment-related outcomes, survival, and prognosis related to primary endometrial and ovarian cancers of different variants.

## Materials and methods

The study was granted approval by the Institutional Review Board (IRB) of Shaukat Khanum Memorial Cancer Hospital and Research Centre. Between 2005 and 2017, we retrieved data relating to 30 patients diagnosed histologically with synchronous endometrial and ovarian tumors from the Cancer Registry database. The diagnosis was established by an expert panel of pathologists at our institution.

Data that were analyzed included age, body mass index (BMI), parity, addiction, presenting complaints, comorbidities like diabetes mellitus (DM) and hypertension (HTN), family history of malignancy, and use of hormones. Details of pathological diagnosis, grading, type, size of the tumor, depth of involvement, lymphovascular and perineural invasion, and lymph node status were collected from histopathology reports. World Health Organization (WHO) committee classification was used for histologic determination, and staging of both ovary and endometrium was devised on the basis of the International Federation of Gynecology and Obstetrics (FIGO) guidelines and the American Joint Committee on Cancer (AJCC) staging manual 8th edition. All data were analyzed by using SPSS Statistics version 22.0 (IBM, Armonk, NY). A chi-squared test was applied to assess the association between the above-mentioned variables.

## Results

**Figure 1:**
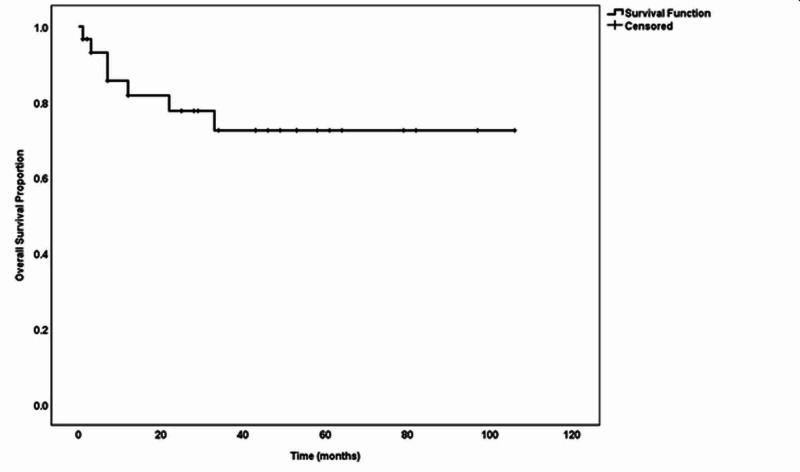
Five-year overall survival

The study included 30 participants with the diagnosis of synchronous endometrial and ovarian cancer treated at Shaukat Khanum Memorial Cancer Hospital and Research Centre, Lahore, Pakistan. The mean age at the time of diagnosis was 51.63 ±11.9 years. Out of the 30 patients, 17 patients were post-menopausal, and half of the female patients were multiparous. The median BMI of the patients was 28.04 kg/m^2^ (range: 17-49 kg/m^2^). The most common presenting complaints were irregular uterine bleeding (30%) followed by post-menopausal bleeding (26.7%) and nonspecific abdominal pain (26.7%). The associated comorbidities included HTN (n=16) followed by DM (n=7) (Table 1). The characteristics features of endometrial and ovarian tumors are presented in Table 2. All of the patients underwent surgical resection; however, among them, 26 patients underwent upfront resection, while four patients were given induction chemotherapy prior to surgical resection. Endometrioid adenocarcinoma was the most common histology at both sites (Table 3). Surgical removal consisted of open (n=26) and laparoscopic (n=2) resections. Adjuvant treatment in the form of platinum-based chemotherapy was given for 25 patients either as concurrent chemotherapy (n=7) or combined chemo-radiotherapy (n=18). Categorical variables such as age, BMI, menopausal, and parity status did not significantly affect five-year survival (Table 4). At the final follow-up, 23 patients were found to be alive with no recurrence, while six had died due to tumor recurrence and metastasis. One patient was lost to follow-up. Figure 1 charts the overall five-year survival (75%).

**Table 1:** Baseline characteristics of synchronous tumor patients

Variables	Categorization	N (%)
Age (years)	Up to 50	11 (36.7)
	Above 50	19 (63.3)
Parity	Nulliparity	15 (50)
	Multiparity	15 (50)
Body mass index	Up to 25 kg/m^2^	07 (23.3)
	Above 25 kg/m^2^	23 (76.7)
Menopausal status	Pre-menopausal	13 (43.3)
	Post-menopausal	17 (56.7)
Marital status	Married	23 (76.7)
	Unmarried	07 (23.3)
Surgical complications	Bleeding	02 (6.7)
	Pulmonary embolism	01 (3.3)
	Infection	01 (3.3)
	Deep vein thrombosis	01 (3.3)
Major complaints	Abdominal pain	08 (26.7)
	Irregular uterine bleeding	09 (30)
	Mass abdomen	05 (16.7)
	Post-menopausal bleeding	08 (26.7)

**Table 2:** Characteristics of endometrial and ovarian cancers

Variables	Categorization	N (%)
Characteristics of endometrial cancer		
Grading	G1	12 (40)
	G2	13 (43)
	G3	5 (16)
Staging	S1	21 (70)
	S2	5 (16.7)
	S3	3 (10)
	S4	1 (3.3)
Lymphovascular invasion	Yes	5 (16.7)
	No	25 (83.3)
Depth of invasion	Above 50%	10 (33.3)
	Up to 50%	1 (3.3)
	Not known	19 (63.3)
Characteristics of ovarian cancer		
Grading	G1	12 (40)
	G2	12 (40)
	G3	6 (20)
Staging	S1	22 (73.3)
	S2	3 (10)
	S3	2 (6.7)
	S4	3 (10)
Lymphovascular invasion	Yes	5 (16)
	No	25 (83.3)

**Table 3:** Tumor histological variants and treatment modalities used TAH: total abdominal hysterectomy; BSO: bilateral salpingo-oophorectomy; PLND: pelvic lymph node dissection

Variables	Categorization	N (%)
Endometrial cancer		
Endometrium tumor histology	Endometrial adenocarcinoma	25 (83.3)
	High-grade serous carcinoma	4 (13.3)
	Undifferentiated endometrial sarcoma	1 (3.3)
Ovarian cancer		
Ovarian tumor histology	Endometrial adenocarcinoma	20 (66.6)
	Mucinous cystadenocarcinoma	5 (16.6)
	Brenner tumor	5 (16.6)
Treatment modalities		
Variables	Categorization	N (%)
Neo-adjuvant treatment	Induction chemotherapy	4 (13.3)
Surgery	TAH + BSO	17 (57)
	TAH + BSO + omentectomy + appendectomy	03 (10)
	TAH + BSO + PLND	10 (33)
Adjuvant treatment	Radiotherapy	18 (60)

**Table 4:** Survival analysis based on categorical variables

Variable	Categorization	Five-year overall survival	P-value
Age (years)	Up to 50	63%	0.14
	Above 50	85%	
Menopausal state	Pre-menopausal	68%	0.28
	Post-menopausal	83%	
Parity	Nulliparous	63%	0.40
	Multiparous	83%	
Body mass index	Up to 25 kg/m^2^	80%	0.96
	Above 25 kg/m^2^	74%	

## Discussion

Synchronous endometrial and ovarian tumors are rare variants of gynecological cancers, and they must be differentiated from either primary endometrium or ovarian tumors with metastasis. The landmark criteria have been laid down by Ulbright and Roth, and later refined substantially by Scully et al. [4,5]. Obesity has resulted in changing trends of endometrial disease in the United States, resulting in a gradual increase in the incidence of the disease in the past few decades [10,11]. Menopausal hormonal therapy as well as the use of oral contraceptives have caused a steady fall in the incidence of ovarian cancer. Many theories have been proposed about these relative proportions of synchronous tumors, which include changing demographic trends and the introduction of diagnostic criteria in 1980, which was adopted by many centers in later years [12-14].

Matsuo et al. have reported a peak incidence in females who are above 40 years of age [15]. Studies have demonstrated an earlier median age of diagnosis for synchronous tumors compared to those developing either endometrial or ovarian alone [6,9,16]. The median age at diagnosis in our cohort was 51 years (range: 25-72 years). Some studies have reported the presence of synchronous tumors in 10-29% of women who are less than 45 years of age [17-19]. Herrinton et al. have reported synchronous tumors in 39% of patients below the age of 50 years [20].

Obesity has been known as a risk factor for endometrial carcinoma although its association with ovarian tumors is not yet established. The risk to develop endometrial cancer increases by three-fold and nine-fold for women having a bodyweight of 9-22 kg and more than 22 kg above the normal limit, respectively [21]. The median BMI was 28.04 kg/m^2^ in our cohort. More than 75% of patients in our series had a BMI of >25 kg/m^2^, and this was consistent with an institutional review by Soliman et al. who have reported obesity in 60% of their patients [9].

Although nulliparity is related to ovarian cancers, Herrinton et al. have reported lower than normal mean parity in women with synchronous tumors as compared to those with either endometrial or ovarian tumor alone [20,22]. We observed nulliparous status in 33% of our patients. Eifel et al. have found an increased (50%) presentation of nulliparous status in the synchronous endometroid/endometroid variant of these tumors [6]. In our series, 45% of patients were nulliparous against the background of endometroid/endometroid combination.

All the above-mentioned factors such as obesity, nulliparity, and comparatively younger age may attribute a “hormonal field effect” to the development of synchronous endometrioid cancers. The morphological unit consisting of the uterus, fallopian tubes, and ovary as part of the müllerian system may explain the synchronous appearance of these malignancies [6]. Niskakoski et al. have suggested a shared origin of synchronous endometrial and ovarian carcinoma in Lynch syndrome (LS), indicating converging pathways of tumorigenesis. L1 cell adhesion molecule (L1CAM) overexpression was significantly common (43%) among synchronous patients [23].

Previous studies have demonstrated improved survival outcomes (approximately 80% over 10 years) in synchronous endometrial and ovarian tumor patients [2,23]. Our cohort demonstrated overall and five-year survival of 75%. We also analyzed survival outcomes based on other factors such as age, parity, pre-/post-menopausal state, and BMI. Interestingly, age above 75 years, multiparity, BMI of less than 25 kg/m^2^, and post-menopausal status showed improved yet not significant survival outcomes (Figure 1).

All patients in our cohort underwent surgical intervention. The standard operation involved hysterectomy and bilateral salpingo-oophorectomy (BSO) with or without removal of pelvic lymph nodes, and completion surgery on the ovarian protocol, which consisted of infracolic omentectomy and peritoneal biopsies and washings. Pelvic lymph node dissection (PLND) as part of the FIGO staging was performed in some patients, but it had no therapeutic benefit other than allocating patients to poor prognostic groups. The most commonly encountered surgery-related complication was bleeding (two patients), while one patient developed a postoperative infection.

Our study has certain limitations, such as its retrospective nature, the small number of patients enrolled due to the rarity of the disease, and a lack of molecular profiling due to cost-related issues in a developing country with poor socioeconomic status. Nevertheless, we believe the results will help toward a better understanding of the trends and outcomes of this rare entity in the South Asian population. Multicenter studies with a larger number of patients may enable us to delineate this subset of patients in a more comprehensive manner in order to provide more focused and tailored treatment.

## Conclusions

In our study, we retrospectively examined 30 patients with synchronous ovarian and endometrial cancers treated at a tertiary care referral center in South Asia. We can conclude that patients with synchronous primary endometrial and ovarian cancers have better overall survival than patients with single primary ovarian or endometrial cancers. Also, synchronous primary endometrial and ovarian cancer endometroid types have better overall survival than patients with non-endometrioid or mixed histologic types.
